# Urinary Sediment Transcriptomic and Longitudinal Data to Investigate Renal Function Decline in Type 1 Diabetes

**DOI:** 10.3389/fendo.2020.00238

**Published:** 2020-04-30

**Authors:** Maria Beatriz Monteiro, Tatiana S. Pelaes, Daniele P. Santos-Bezerra, Karina Thieme, Antonio M. Lerario, Sueli M. Oba-Shinjo, Ubiratan F. Machado, Marisa Passarelli, Suely K. N. Marie, Maria Lúcia Corrêa-Giannella

**Affiliations:** ^1^Laboratório de Carboidratos e Radioimunoensaio (LIM-18), Faculdade de Medicina, Hospital das Clinicas HCFMUSP, Universidade de São Paulo, São Paulo, Brazil; ^2^Department of Physiology and Biophysics, Institute of Biomedical Sciences, University of São Paulo, São Paulo, Brazil; ^3^Division of Metabolism, Endocrinology, and Diabetes, Department of Internal Medicine, University of Michigan, Ann Arbor, MI, United States; ^4^Laboratory of Molecular and Cellular Biology (LIM-15, Faculdade de Medicina, Hospital das Clinicas HCFMUSP, Universidade de São Paulo, São Paulo, Brazil; ^5^Laboratório de Lípides (LIM-10), Faculdade de Medicina, Hospital das Clinicas HCFMUSP, Universidade de São Paulo, São Paulo, Brazil; ^6^Programa de Pós-graduação em Medicina, Universidade Nove de Julho (UNINOVE), São Paulo, Brazil

**Keywords:** diabetic kidney disease, transcriptomics, renal function decline, longitudinal data, type 1 diabetes, urine

## Abstract

**Introduction:** Using a discovery/validation approach we investigated associations between a panel of genes selected from a transcriptomic study and the estimated glomerular filtration rate (eGFR) decline across time in a cohort of type 1 diabetes (T1D) patients.

**Experimental:** Urinary sediment transcriptomic was performed to select highly modulated genes in T1D patients with rapid eGFR decline (decliners) vs. patients with stable eGFR (non-decliners). The selected genes were validated in samples from a T1D cohort (*n* = 54, mean diabetes duration of 21 years, 61% women) followed longitudinally for a median of 12 years in a Diabetes Outpatient Clinic.

**Results:** In the discovery phase, the transcriptomic study revealed 158 genes significantly different between decliners and non-decliners. Ten genes increasingly up or down-regulated according to renal function worsening were selected for validation by qRT-PCR; the genes *CYP4F22*, and *PMP22* were confirmed as differentially expressed comparing decliners vs. non-decliners after adjustment for potential confounders. *CYP4F22, LYPD3, PMP22, MAP1LC3C, HS3ST2, GPNMB, CDH6*, and *PKD2L1* significantly modified the slope of eGFR in T1D patients across time.

**Conclusions:** Eight genes identified as differentially expressed in the urinary sediment of T1D patients presenting different eGFR decline rates significantly increased the accuracy of predicted renal function across time in the studied cohort. These genes may be a promising way of unveiling novel mechanisms associated with diabetic kidney disease progression.

## Introduction

Diabetic kidney disease (DKD) is a major cause of end-stage renal disease (ESRD) worldwide. The prevalence of ESRD in patients with diabetes (DM) is up to 10 times higher than those without DM (1). The search for both prognostic and surrogate endpoint biomarkers for DKD has received more attention in the recent years. However, at present no novel biomarkers are routinely used in the clinic or in trials (2).

Recently, the use of urine in the study of DKD has been increasing with the use of quantitative polymerase chain reaction (PCR) or ELISA (3). In the last decade, next generation sequencing (NGS) methods made it possible to generate inexpensive, reproducible, and high throughput nucleic acid sequence data providing new opportunities for unbiased discovery of novel pathophysiologic pathways of disease, as well as for the identification of novel disease biomarkers (4).

Besides being an organ-specific sample, urine is an easily obtainable material, without the need of invasive procedures. We previously described the expression of markers of proximal tubule epithelial cells and podocytes in the human urine sediment while attempting to correlate clinical markers of kidney disease with expression of target genes in this biological material (5). The same approach was taken by other research groups (3) showing the use of urine as a translatable strategy to study markers of kidney injury without the need of invasive interventions.

In an attempt to identify new pathways associated with DKD progression, we performed a transcriptomic analysis using total RNA isolated from urine sediment cells collected from patients with type 1 DM (T1D), presenting or not rapid renal function decline. Candidate genes identified in the transcriptomic study were validated in a cohort of T1D patients followed from 2006 to 2018. Eight of the 10 validated genes significantly increased the accuracy of predicted renal function across time in the studied cohort. These genes may be a promising way of unveiling novel mechanisms associated with DKD progression.

## Materials and Methods

### Participants

This study was conducted in compliance with the Institutional Ethics Committee and the Declaration of Helsinki of 1975, revised in 1983, with informed consent being given to all participants and it was approved by the Ethics Committee of the University of São Paulo Medical School (Cappesq, approval #1,536,656). Fifty-four T1D patients were recruited from 2012 to 2016 at the Diabetes Outpatient Clinic, Hospital das Clinicas da Faculdade de Medicina da Universidade de São Paulo, Brazil. T1D was diagnosed in patients presenting hyperglycaemia, positive autoantibodies [glutamic acid decarboxylase [GAD], islet cell antibodies or tyrosine phosphatase-like insulinoma antigen 2 [IA-2]], undetectable C-peptide or ketoacidosis and insulin requirement within 3 months after diagnosis. All patients were receiving intensive insulin therapy. At the time of recruitment, urine samples were collected as previously described (5, 6) and later used for gene expression analyses. Briefly, urine specimens were collected in sterile RNAse-free flasks. All samples were submitted to urinalysis test and the samples testing positive for leucocyturia (>10,000 leucocytes per cubic millimeter of urine) were discarded. Total RNA was isolated using Trizol reagent (ThermoFisher Scientific, Carlsbad, CA) and RNeasy Minikit (Qiagen, Germantown, MD) followed by reverse-transcription to cDNA using High Capacity cDNA Reverse Transcription Kit (ThermoFisher Scientific, Carlsbad, CA).

Clinical and demographic data were collected starting from the date each patient was admitted to the Diabetes Clinic and ending in 2018 or until reaching end-stage renal disease (median follow-up of 12 years; mean±SD follow-up of 11 ± 2.9 years). Data collection included sex, age, T1D duration, use of angiotensin converting enzyme inhibitors (ACEi) or angiotensin receptor blocker (ARB), and glycated hemoglobin (HbA1c) values. To evaluate renal function across time, we collected all measurements for serum creatinine taken during the entire follow-up period (at least one per year of follow-up, median of 3 measurements per year). Serum creatinine was measured by the Jaffé reaction, standardized to IDMS traceable creatinine and used to calculate the estimated glomerular filtration rate (eGFR) using the Chronic Kidney Disease Epidemiology Collaboration (CKD-EPI) equation (7).

### Library Preparation and RNA Sequencing

Four T1D patients with an eGFR decline ≥3.5 mL/min/1.73 m^2^ per year of follow-up (decliners) eGFR, four T1D patients with an eGFR decline <3.5 mL/min/1.73 m^2^ per year of follow-up (non-decliners), and two non-diabetic controls were selected for the transcriptomic study. Messenger RNA was checked for quality (RIN value >7.0) and quantity using Agilent 2200 TapeStation (Agilent Technologies) employing the High Sensitivity D1000 ScreenTape assay. RNA-seq libraries were prepared using Illumina TruSeq Stranded Total RNA Library Prep Kit with Ribo-Zero Gold® (San Diego, CA) from 100 ng of purified total RNA according to the manufacturer's protocol. Paired-end reads were generated in the Illumina HiSeq 2000 platform. The dataset is available with National Center for Biotechnology Information's Gene Expression Omnibus database under the accession number GSE140627.

### Bioinformatic Analysis

Reads were aligned to the hg38 version of the human reference genome (downloaded from ftp://ftp.ensembl.org/pub/release-94/fasta/homo_sapiens/dna/ using STAR (8). Metrics such as number of reads, duplicate levels, total number of expressed genes and ribosomal RNA contamination were generated with RNASeqC (9). Count data was generated with featureCounts (10). Non-expressed genes were flagged and removed from downstream analysis using *DAFS* (11). Data normalization and logCPM transformation was performed using the *voom* function from the R/Bioconductor tool *limma* (11). Differential gene expression analysis was performed using *limma*. Pairwise comparisons between control samples and diabetic samples (decliners and non-decliners) were performed. Gene set enrichment analysis was performed with *WebGestalt* (12).

### qRT-PCR Validation

Urinary sediment total RNAs from 54 T1D patients were used to validate the results of the transcriptomic study. Messenger RNA was checked for quality (RIN value >5.0 for the samples used in the validation phase). Starting from the lowest value of *p*, the top 10 genes that were increasingly up or down-regulated according to renal function worsening were selected for validation by qRT-PCR. Gene expression analyses were performed with 10 ng of cDNA/sample, in duplicates, with the use of Taqman assays in a StepOne plus Real-Time PCR System (ThermoFisher Scientific, Carlsbad, CA). The relative mRNA abundance was calculated using the 2–ΔΔCt method (13) and E74-Like Factor 1 (ELF1) was used as reference gene (5). Taqman assays are listed in Supplemental Table 1.

### Statistical Analyses

Statistical analyses were performed by JMP Pro version 13.0 (SAS Institute, Cary, NC). Before the analyses, normalized mRNA expression values were log_10_ transformed. Non-parametric Wilcoxon/Kruskal Wallis test followed by Tukey-Kramer's multiple comparison test were used to identify the differences among decliners, non-decliners and controls in the cross-sectional analyses; analyses between T1D patients classified as decliners and non-decliners were adjusted by sex, diabetes duration, body mass index (BMI), use of ACEi or ARB, Hba1c, urinary albumin excretion, and creatinine (to account for the stage of DKD at the beginning of the follow-up) at the time of the urine collection.

Linear mixed-effects models (random-effects models) were used to test the association between each of the genes and the longitudinal change in eGFR during the follow-up period. Sex, diabetes duration, BMI, use of ACEi or ARB, HbA1c, urinary albumin excretion (all at the time of the urine collection) and follow-up time and its interaction with the normalized expression of each gene were used as fixed effects. Additionally, as measurements of the same patients were taken repeatedly through time, we used patient's identification as a random effect, which enabled to account for: differences on the initial eGFR values, within-individual changes of eGFR overtime and correlation among repeated measurements on the same patient. Each gene was evaluated separately. Patients with an initial eGFR <15 mL/min/1.73 m^2^ were excluded from the linear mixed-effects analyses. A *P* < 0.05 was considered statistically significant.

## Results

### Pathways Modulated in Patients With Rapid Renal Function Decline

Clinical characteristics and renal function evolution of the patients selected for the transcriptomic study are presented in the Supplemental Table 2 and in the Supplemental Figure 1, respectively. Quality control data for the RNA sequencing protocol is shown in the Supplemental Table 3; three samples showing low reads were excluded and the seven remaining samples showed between 16 and 25 million reads.

**Figure 1:**
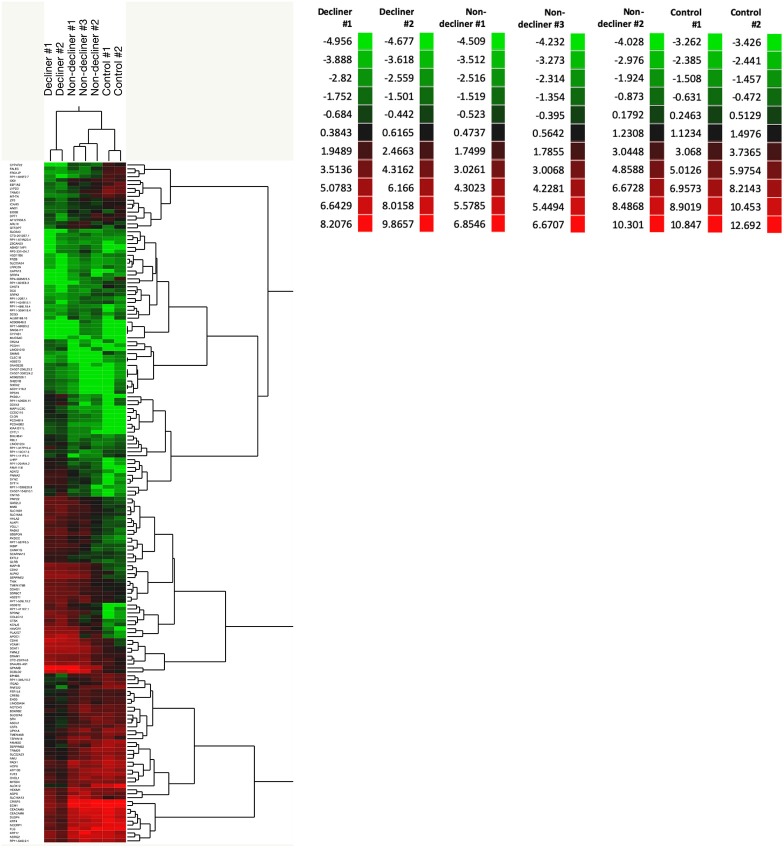
A total of 158 genes were differentially expressed between decliners vs. non-decliners. Expression profile of differentially expressed genes in type 1 diabetes patients (decliners and non-decliners) and in healthy non-diabetes controls analyzed by hierarchical clustering (2- way clustering, Ward method). Row: single gene; column: urinary sediment sample. Color legends showing normalized gene expression levels for each patient.

**Figure 2:**
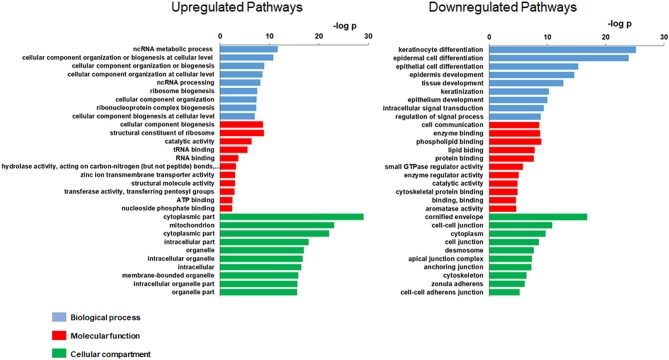
Gene Ontology (GO) of the modulated transcripts in patients with rapid renal function decline. Gene Ontology categories of the modulated transcripts from type 1 diabetes patients classified as decliners vs. non-decliners.

**Figure 3:**
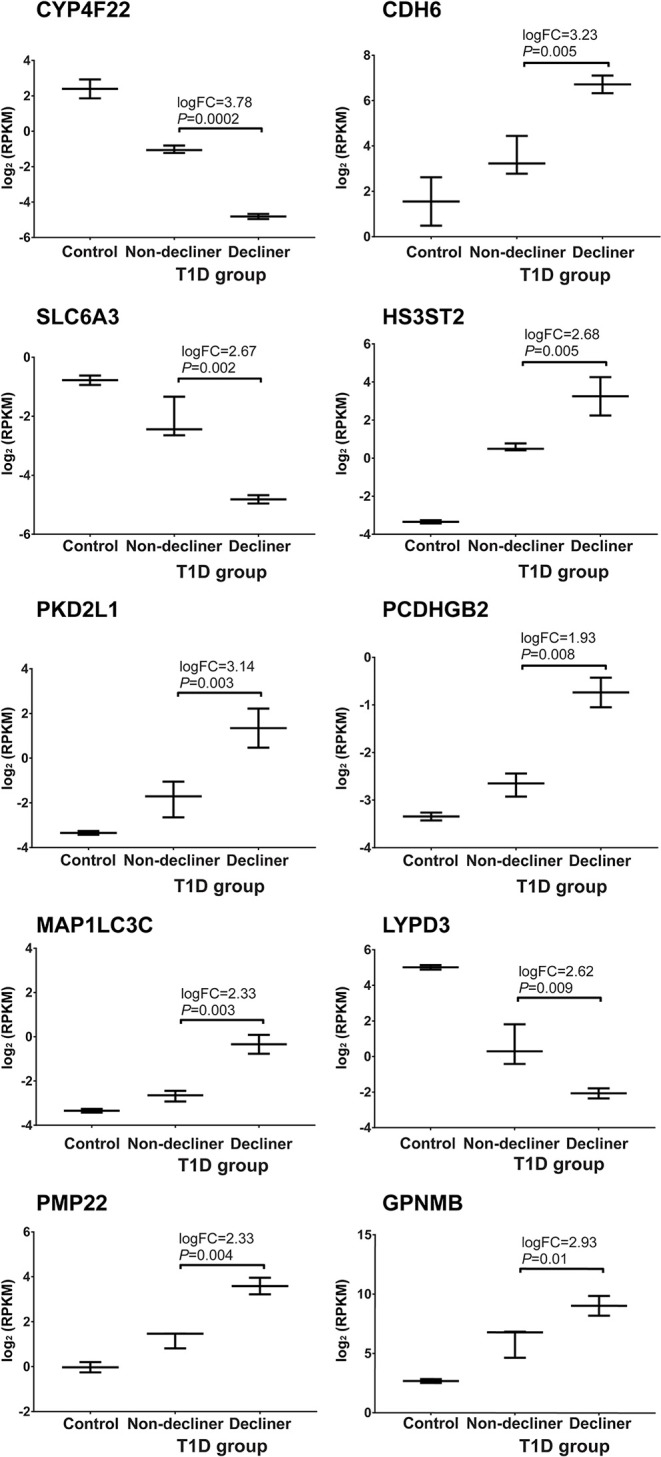
Top 10 genes progressively modulated selected for validation. RNA sequencing expression levels of the top 10 genes increasingly up- or down-regulated according to renal function worsening selected for validation by qRT-PCR. Bars representing median value and interquartile range. T1D, type 1 diabetes.

A total of 158 genes were differentially expressed between decliners vs. non-decliners; 73 up-regulated and 85 down-regulated (log fold-change >1.5 and < -1.5, respectively; *P* < 0.05) (Supplemental Table 4). Hierarchical clustering performed for the differentially expressed genes resulted in the dendrogram shown in Figure 1. The classification of the transcripts up or down-regulated in decliners vs. non-decliners according to Gene ontology (GO) categories is shown in Figure 2. Figure 3 elicits the RNA sequencing expression levels of the 10 genes selected for validation by qRT-PCR: Cytochrome P450 family 4 subfamily F member 22 (*CYP4F22*), Solute carrier family 6 member 3 (*SLC6A3*), Polycystin 2 like 1, transient receptor potential cation channel (*PKD2L1*), Microtubule associated protein 1 light chain 3 gamma (*MAP1LC3C*), Peripheral myelin protein 22 (*PMP22*), Cadherin 6 (*CDH6*), Heparan sulfate-glucosamine 3-sulfotransferase 2 (*HS3ST2*), Protocadherin gamma subfamily B, 2 (*PCDHGB2*), LY6/PLAUR domain containing 3 (*LYPD3*), and Glycoprotein nmb (*GPNMB*).

### Validation of Genes Associated With Rapid Renal Function Decline

In order to validate the findings from the transcriptomic study, we used qRT-PCR and mRNA samples from 54 T1D patients classified as decliners and non-decliners as described above. Patients were mostly female (66% women), 34 [28–41.5] years old (median [interquartile interval]), 22 [16–29] years of diabetes duration, age at diagnosis of 13 [7–17] years old, with an HbA1c of 8.1% [7.2–9] and 65.7% had eGFR ≥60 mL/min/1.73 m^2^ at the time of urine collection. There were no differences between decliners and non-decliners regarding age at diagnosis, HbA1c and use of ACEi, ARB or statins. Non-decliners had longer DM duration (25 ± 10 vs. 20 ± 7 years, respectively) than decliners at the time of urine collection. We also included 12 healthy non-diabetes controls [61% women, 32 [24.2–53.2] years old] with no history of DM or renal disease.

**Figure 4:**
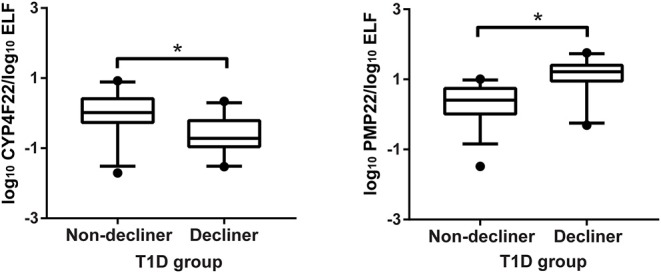
Validation of two genes associated with rapid renal function decline. Cross-sectional validation of genes differentially expressed in human urinary sediment cells from type 1 diabetes (T1D) patients classified as non-decliners or decliners (eGFR < or ≥3.5 mL/min/1.73 m^2^ per year of follow-up, respectively). Analyses adjusted by sex, diabetes duration, body mass index, use of angiotensin converting enzyme inhibitors or angiotensin receptor blocker, HbA1c, urinary albumin excretion, and creatinine at the time of the urine collection. Bars representing median value and interquartile range. *P < 0.05.

*SLC6A3* and *PCDHGB2* displayed late amplification curves in several samples and were excluded from further analyses. Cross-sectional analyses revealed significant modulation of the genes *CYP4F22, LYPD3, PMP22*, and *MAP1LC3C* between controls and T1D patients classified as decliners and non-decliners (Supplemental Figure 2). When only T1D patients were considered, up-regulation of the genes *MAP1LC3C* (*P* < 0.001), *CDH6* (*P* = 0.02), *GPNMB* (*P* = 0.009), *HS3ST2* (*P* = 0.01), *PMP22* (*P* < 0.001), *and PKD2L1* (*P* = 0.04), and down-regulation of the genes *CYP4F22* (*P* < 0.001) and *LYPD3* (*P* = 0.01) were observed in decliners in comparison to non-decliners (Supplemental Figure 3). After adjustment for potential confounders, only *CYP4F22* and *PMP22* were significantly modulated between decliners and non-decliners (Figure 4).

### Eight Out of the Ten Validated Genes Significantly Modified the Slope of eGFR

We next sought to investigate if the genes selected for validation could improve the estimation of the longitudinal changes in eGFR during the follow-up period performing a linear mixed-effects model for each gene. Eight genes significantly modified the slope of eGFR in T1D patients across time: *CYP4F22, LYPD3, PMP22, MAP1LC3C, HS3ST2, GPNMB, CDH6*, and *PKD2L1* (Table 1).

**Table 1:** Linear mixed model estimates ± standard error (SE) for the expression of genes which significantly modify the slope of estimated glomerular filtration rate in Type 1 diabetes patients across time.

**Gene symbol**	**Estimate**	**SE**	***P* value**
*CYP4F22*	0.0053	0.0005	<0.0001
*LYPD3*	0.0080	0.001	<0.0001
*PMP22*	−0.0159	0.0009	<0.0001
*MAP1LC3C*	−0.0137	0.001	<0.0001
*HS3ST2*	−0.0120	0.0009	<0.0001
*GPNMB*	−0.0094	0.0008	<0.0001
*CDH6*	−0.0076	0.0006	<0.0001
*PKD2L1*	−0.0073	0.0009	<0.0001

## Discussion

In an attempt to identify new pathways associated with kidney function decline in the setting of DKD, we performed transcriptomic analyses of urinary sediment cells obtained from T1D patients presenting different eGFR decline rates. We validated the findings from the transcriptomics study in a cohort of patients followed longitudinally and eight out of the 10 validated genes significantly modified the slope of eGFR across time, adding prognostic value beyond established risk factors.

Most of the identified genes have never been associated with changes in kidney function, becoming interesting potential new targets for the study of DKD. Pathways related to most of them, however, have already been associated with kidney diseases, including DKD.

*HS3ST2* encodes an isoform of heparan sulfate 3-O sulfotransferase, an enzyme involved in heparan sulfate (HS) biosynthesis. Not only abnormal metabolism of HS has been reported in DKD (14), but also variants in a gene encoding another HS-O sulfotransferase (*HS6ST1*) were associated with albuminuria in type 2 diabetes patients (15).

The *PMP22* gene, also known as *GAS3* (growth-arrest-specific protein 3), encodes a glycoprotein whose mutations cause neuropathy-related diseases and whose functions remain incompletely known (16). Besides being a constituent of peripheral nerve myelin, PMP22 is also involved in cell-cell junctions; in wounded kidney epithelial cells (MDCK cells), the overexpression of PMP22 decreased proliferation and migration and altered permeability of cell monolayers (17). It is worth mentioning that myelin protein 0 (MPZ or P0), the major component of myelin, is expressed in human and rodent podocytes and plays an important role in glomerular permeability, since increased urinary albumin excretion was shown in mice deficient for this protein (18).

*CYP4F22* encodes a member of the cytochrome P450 superfamily (19); cytochrome P450 4F isoforms metabolize arachidonic acid to generate 20-hydroxyeicosatetraenoic acid (20-HETE) (20). This reaction is thought to be catalyzed by CYP4F2 in the kidneys, where 20-HETE acts as a natriuretic and vasoactive eicosanoid and participates in the control of renal function and systemic blood pressure (21, 22). Altered renal 20-HETE content was related to hypertension in animal models and in humans (23–25). However, little is known about the regional distribution of renal CYP4Fs (24), which includes CYP4F22. This isoform had been identified as one of the autosomal recessive congenital ichthyosis-causative genes (26). Nonetheless *CYP4F22* and *CYP4F2* are described as paralogs and genetic variants in the *CYP4F2* were associated with hypertension (23). We observed decreased expression of *CYP4F22* mRNA in the decliner group; downregulation of *CYP4A11* mRNA, another CYP4 isoform involved in 20-HETE generation, was already described in a gene expression profile of renal biopsies from patients with hypertensive nephropathy (27).

*MAP1LC3C* encodes a key structural protein of the autophagosome considered a marker of autophagy, as are the other members of the LC3 family *MAP1LC3A* and *MAP1LC3B* (28). Impaired autophagy in glomerular and tubular cells has been recognized in the pathogenesis of DKD, contributing to the accumulation of cellular damage (29). A decreased expression of *MAP1LC3A* mRNA was described in the urinary sediment from patients with DKD (30), an opposite finding to what we observed in the present study for *MAP1LC3C*. However, it is not clear whether the three LC3 proteins have the same biological function in autophagy or in other pathways (28). The potential participation of *MAP1LC3C* in DKD is corroborated by the finding of a variant in this gene conferring susceptibility to eGFR decline over time in a genome–wide association study in European American participants of the Chronic Renal Insufficiency Cohort Study (31).

We were not able to find studies reporting the participation of *LYPD3* (LY6/PLAUR Domain Containing 3, also known as *C4.4A*), a urokinase-type plasminogen activator receptor (uPAR) homolog, in kidney diseases. In an immortalized non-tumorigenic human epidermal cell line (HaCaT), decreased expression of LYPD3 was detected after induction of epithelial–mesenchymal transition by TGFβ (32), a process already associated with tubulointerstitial fibrosis in diabetic nephropathy (33). We found no studies reporting the participation of *PKD2L1*, a member of the polycystin protein family involved in cell-cell/matrix interactions, in DKD.

GPNMB is a transmembrane glycoprotein expressed on renal tubular cells and on cells of the monocyte–macrophage lineage (34). This protein was already described as a biomarker of progressive renal injury; its increased expression was found in the kidney of rats with streptozotocin-induced diabetes and in kidney and urine of patients with progressive kidney disease, including DKD (35). Following renal ischemic damage, GPNMB expression increases in macrophages and in surviving epithelial cells and it is required for phagocytosis, recruitment of members of the LC3 family, and, eventually, autophagy and tissue repair (36). Urinary concentrations of GPNMB failed to confer prognostic value for renal function decline beyond established risk factors in patients with type 2 diabetes, despite correlating with the severity of albuminuria (34). However, in the present study, *GPNMB* mRNA expression in the urinary sediment modified the slope of eGFR in T1D patients across time, maybe reflecting a compensatory mechanism of tissue repairing as kidney disease progresses.

CDH6 or kidney cadherin belongs to the cadherin superfamily of cell surface glycoproteins essential to tissue development and to cell-cell adhesion (37). In a transcriptional analysis of human kidney organoids derived from pluripotent stem cells, *CDH6* mRNA was identified as highly expressed in immature glomerular epithelial cells and reactivated in injured podocytes in chronic kidney diseases, including DKD. Renal expression of this gene was also associated with proteinuria and with loss of renal function in cohort of patients with kidney disease (38).

To our knowledge, this is the first study evaluating the urinary sediment transcriptome in the setting of DKD. Strengths of our study include the longitudinal study design and the use of multiple values of eGFR across time, providing an accurate analysis of kidney function evolution for each patient. The main limitations are associated to the low number of patients in the discovery and validation phases. In the transcriptomic study we initially included 10 samples but analyzed only seven due to insufficient coverage to secure diversity of transcripts. Differential gene expression is presented by nominal *P* values instead of adjusted *P* values as a result of the low sample number. In the validation phase, the difficulties of obtaining good quality mRNA from urinary sediment resulted in a relatively small number of patients and the lack of replication in an independent cohort.

In summary, genes selected from a transcriptomic analysis of the urinary sediment increased the accuracy of predicted renal function across time in the studied cohort of T1D patients. Some of the genes identified as differentially expressed between two groups presenting distinct eGFR decline rates corroborated the involvement of pathways previously associated to DKD, such as abnormal metabolism of HS and of 20-HETE, autophagy, as well as the participation of compensatory tissue repair as kidney disease progresses.

## Data Availability Statement

The datasets generated for this study can be found in the NIH/GEO GSE140627.

## Ethics Statement

The studies involving human participants were reviewed and approved by Ethics Committee of the University of São Paulo Medical School (Cappesq, approval #1,536,656). The patients/participants provided their written informed consent to participate in this study.

## Author Contributions

MM, TP, DS-B, and KT collected samples & clinical data and analyzed the results. AL, SO-S, and SM performed the RNA sequencing study and analyzed the transcriptomic data. MC-G, MP, and UM designed the study and provided the funds. MC-G and MM wrote the manuscript.

## Conflict of Interest

The authors declare that the research was conducted in the absence of any commercial or financial relationships that could be construed as a potential conflict of interest.
