# Supplementary material for: Urinary Sediment Transcriptomic and Longitudinal Data to Investigate Renal Function Decline in Type 1 Diabetes
Source: Front Endocrinol (Lausanne). 2020 Apr 30;11:238. doi: 10.3389/fendo.2020.00238 (PMC7204506; doi:10.3389/fendo.2020.00238)
Supplement: Supplementary file 1 [file Data_Sheet_1.PDF]

SUPPLEMENTAL MATERIAL

Table of contents **Page**

1. **Supplemental Tables**

   1.1 - Supplemental Table 1. Gene expression assays used in qPCR reactions in the validation study 2

   1.2 - Supplemental Table 2. Clinical characteristics of the patients selected for the transcriptomics study 3

   1.3 - Supplemental Table 3: Quality control data for the RNA sequencing protocol 4

   1.4 - Supplemental Table 4: Genes differentially expressed between type 1 diabetes patients classified as decliners or non-decliners 5

2. **Supplemental Figures**

   2.1 - Supplemental Figure 1. Estimated glomerular filtration rate (eGFR) evolution of the type 1 diabetes patients selected for the transcriptomics study. 11

   2.2 - Supplemental Figure 2. Four genes differentially expressed in non-diabetic controls and type 1 diabetes (T1D) patients classified as non-decliners or decliners. 12

   2.3 - Supplemental Figure 3. Eight genes differentially expressed between type 1 diabetes (T1D) patients classified as non-decliners or decliners without adjustment for possible confounders**.** 13

1. Supplemental Tables

Supplemental Table 1. Gene expression assays used in qPCR reactions in the validation study

Gene	Assay ID
CYP4F22	Hs00403446_m1
SLC6A3	Hs00997374_m1
PKD2L1	Hs00175850_m1
MAP1LC3C	Hs01374916_m1
PMP22	Hs00165556_m1
CDH6	Hs01026780_m1
HS3ST2	Hs00428644_m1
PCDHGB2	Hs00251715_m1
LYPD3	Hs01012111_m1
GPNMB	Hs01095669_m1

Supplemental Table 2. Demographic and clinical characteristics of the patients selected for the transcriptomics study

Sample	Sex	Age (years)	T1D duration (years)	Follow-up time (years)	Initial eGFR	Final eGFR	Urinary ACR (mg/g creatinine)
Non-Decliner #1	Female	38	25	4	22	21	> 300
Non-Decliner #2	Male	48	21	13	114	78	> 300
Non-Decliner #3	Male	50	33	12	100	68	30 - 300
Decliner #1	Male	25	16	1.1	51	19	> 300
Decliner #2	Male	43	17	4.1	39	11	> 300
Control #1	Female	27	-	-	-	-	-
Control #2	Female	33	-	-	-	-	-
Decliner #3 EXCLUDED	Male	22	17	2.2	84	40	> 300
Decliner #4 EXCLUDED	Female	38	21	2.2	75	58	< 30
Non-Decliner #4 EXCLUDED	Female	30	29	2.1	77	75	30-300

T1D – type 1 diabetes mellitus, eGFR – estimated glomerular filtration rate, ACR – albumin to creatinine ratio

Supplemental Table 3: Quality control data for the RNA sequencing protocol

Sample	Total Purity Filtered Reads Sequenced	Intragenic Rate	Exonic Rate	Intronic Rate	Intergenic Rate	Expression Profiling Efficiency	rRNA rate	Transcripts Detected	Genes Detected
Non Decliner #1	30,685,504	0.921	0.679	0.242	0.079	0.513	0.270	113.956	20.350
Non Decliner #2	23,036,704	0.959	0.854	0.106	0.040	0.798	0.550	122.887	20.222
Non Decliner #3	26,619,646	0.959	0.884	0.075	0.041	0.800	0.611	118.099	19.379
Decliner #1	28,245,426	0.955	0.828	0.126	0.045	0.750	0.523	123.784	20.620
Decliner #2	27,612,472	0.899	0.689	0.210	0.101	0.617	0.359	131.370	23.791
Control #1	23,726,786	0.975	0.886	0.088	0.025	0.705	0.490	96.053	16.514
Control #2	28,446,024	0.856	0.621	0.235	0.144	0.471	0.137	95.953	19.565
Decliner #3 EXCLUDED	43,560,166	0.866	0.482	0.384	0.134	0.019	0.004	39.638	7.865
Decliner #4 EXCLUDED	45,602,716	0.943	0.718	0.225	0.057	0.018	0.010	34.475	7.121
Non Decliner #4 EXCLUDED	21,894,600	0.897	0.583	0.313	0.103	0.025	0.013	28.545	6.311

Supplemental Table 4: Genes differentially expressed between type 1 diabetes patients classified as decliners or non-decliners

Ensembl Gene ID	Gene symbol	Description	logFC Decliner vs Non Decliner	P value
ENSG00000108839	*ALOX12*	arachidonate 12-lipoxygenase	-4.4503612	0.03447863
ENSG00000143631	*FLG*	filaggrin	-4.215108	0.02672097
ENSG00000096006	*CRISP3*	cysteine-rich secretory protein 3	-4.140766	0.01333841
ENSG00000171954	*CYP4F22*	cytochrome P450, family 4, subfamily F, polypeptide 22	-3.7858359	0.00025082
ENSG00000189051	*RNF222*	ring finger protein 222	-3.4979911	0.03168788
ENSG00000120875	*DUSP4*	dual specificity phosphatase 4	-3.4446505	0.02038495
ENSG00000143369	*ECM1*	extracellular matrix protein 1	-3.3885416	0.02911073
ENSG00000105388	*CEACAM5*	carcinoembryonic antigen-related cell adhesion molecule 5	-3.3339863	0.01972394
ENSG00000182580	*EPHB3*	EPH receptor B3	-3.2007149	0.00590593
ENSG00000137699	*TRIM29*	tripartite motif containing 29	-3.1524998	0.04179578
ENSG00000086548	*CEACAM6*	carcinoembryonic antigen-related cell adhesion molecule 6 (non-specific cross reacting antigen)	-3.1350229	0.02055993
ENSG00000118322	*ATP10B*	ATPase, class V, type 10B	-3.1118423	0.02359864
ENSG00000170477	*KRT4*	keratin 4, type II	-3.1107406	0.02322718
ENSG00000131203	*IDO1*	indoleamine 2,3-dioxygenase 1	-3.0951427	0.00597051
ENSG00000197632	*SERPINB2*	serpin peptidase inhibitor, clade B (ovalbumin), member 2	-3.0510793	0.03641305
ENSG00000236740	*RP4-569M23.5*		-3.0153701	0.04489003
ENSG00000215182	*DPF1*	mucin 5AC, oligomeric mucus/gel-forming	-3.0047343	0.04843816
ENSG00000101447	*FAM83D*	family with sequence similarity 83, member D	-2.9375554	0.01545214
ENSG00000214797	*MT-TN*		-2.9039336	0.04730699
ENSG00000171124	*FUT3*	fucosyltransferase 3 (galactoside 3(4)-L-fucosyltransferase, Lewis blood group)	-2.8708475	0.03349962
ENSG00000188505	*NCCRP1*	non-specific cytotoxic cell receptor protein 1 homolog (zebrafish)	-2.8565349	0.03812747

ENSG00000204616	*TRIM31*	tripartite motif containing 31	-2.7803335	0.01931803
ENSG00000101210	*EEF1A2*	eukaryotic translation elongation factor 1 alpha 2	-2.6953415	0.02769259
ENSG00000142319	*SLC6A3*	solute carrier family 6 (neurotransmitter transporter), member 3	-2.6732127	0.00297193
ENSG00000204323	*SFN*	small integral membrane protein 5	-2.6619541	0.04871884
ENSG00000124466	*LYPD3*	LY6/PLAUR domain containing 3	-2.6286411	0.00920701
ENSG00000019102	*SLC22A23*	V-set and immunoglobulin domain containing 2	-2.6100731	0.04624827
ENSG00000183734	*AL592188.10*	achaete-scute family bHLH transcription factor 2	-2.5920706	0.04907318
ENSG00000165795	*NDRG2*	NDRG family member 2	-2.5909555	0.03584161
ENSG00000128422	*KRT17*	keratin 17, type I	-2.5468915	0.02993782
ENSG00000142623	*PADI1*	peptidyl arginine deiminase, type I	-2.521159	0.01950412
ENSG00000210135	*RP3-331H24.7*	mitochondrially encoded tRNA asparagine	-2.4919277	0.04732415
ENSG00000227038	*AF127936.5*	general transcription factor IIi pseudogene 7	-2.4811682	0.04911474
ENSG00000102243	*ASCL2*	vestigial-like family member 1	-2.4510206	0.04907087
ENSG00000109255	*RP11-849F2.7*	neuromedin U 7859	-2.4480336	0.04941337
ENSG00000198574	*NMU*	SH2 domain containing 1B	-2.4137434	0.04929458
ENSG00000274455	*AC009948.5*		-2.3905471	0.04668567
ENSG00000145063	*RP11-823E8.3*		-2.3905471	0.0497801
ENSG00000175315	*CST6*	cystatin E/M	-2.3671854	0.04149025
ENSG00000077279	*DCX*	doublecortin	-2.3032249	0.0299757
ENSG00000074181	*NOTCH3*	notch 3	-2.2772756	0.01306347
ENSG00000102359	*RP11-346J10.2*	sushi-repeat containing protein, X-linked 2	-2.2723448	0.04655307
ENSG00000261504	*CAPN13*		-2.2696904	0.04753423
ENSG00000174514	*MFSD4*	major facilitator superfamily domain containing 4	-2.2564485	0.04285755
ENSG00000105668	*UPK1A*	uroplakin 1A	-2.2448276	0.01557396
ENSG00000263809	*SFRP4*		-2.2413941	0.04947078
ENSG00000272574	*RP11-434B12.1*		-2.2203495	0.04978771
ENSG00000168398	*BDKRB2*	bradykinin receptor B2	-2.2166129	0.02527613
ENSG00000156453	*RP11-640L9.1*	protocadherin 1	-2.2119969	0.04889008
ENSG00000228126	*FALEC*	focally amplified long non-coding RNA in epithelial cancer	-2.1989025	0.04089908

ENSG00000278354	*GTF2IP7*		-2.19476	0.04908102
ENSG00000101276	*SLC52A3*	solute carrier family 52 (riboflavin transporter), member 3	-2.1647636	0.03701933
ENSG00000252481	*TSPAN18*	small Cajal body-specific RNA 13	-2.1340452	0.0468328
ENSG00000025423	*HSD17B6*	hydroxysteroid (17-beta) dehydrogenase 6	-2.1263867	0.01686704
ENSG00000172818	*OVOL1*	ovo-like zinc finger 1	-2.1198837	0.03091257
ENSG00000242741	*RP11-680B3.2*		-2.1078814	0.0491918
ENSG00000240521	*SMG6-IT1*		-2.1078814	0.0491918
ENSG00000278941	*CYP4B1*	SMG6 intronic transcript 1	-2.1078814	0.0491918
ENSG00000174327	*EID2B*	solute carrier family 16, member 13	-2.0974789	0.0498405
ENSG00000140835	*CHST4*	carbohydrate (N-acetylglucosamine 6-O) sulfotransferase 4	-2.0893762	0.01124261
ENSG00000171476	*HOPX*	HOP homeobox	-2.0830769	0.02736497
ENSG00000275374	*ABHD17AP1*		-2.0035591	0.04664816
ENSG00000198658	*CTD-2012B7.1*	abhydrolase domain containing 17A pseudogene 1	-2.0035591	0.04664816
ENSG00000105193	*SLC16A13*	ribosomal protein S16	-1.9323843	0.04982315
ENSG00000188372	*ZP3*	zona pellucida glycoprotein 3 (sperm receptor)	-1.9301273	0.01614032
ENSG00000246526	*SRPX2*		-1.8854066	0.04646648
ENSG00000281491	*RP11-674N23.4*	DNAJB5 antisense RNA 1 (head to head)	-1.8827024	0.04882609
ENSG00000186834	*HEXIM1*	hexamethylene bis-acetamide inducible 1	-1.8767237	0.03474821
ENSG00000267598	*MUC5AC*		-1.8622262	0.04802259
ENSG00000279727	*ZSCAN23*		-1.843721	0.04889926
ENSG00000269416	*SCG3*	long intergenic non-protein coding RNA 1224	-1.8359009	0.04866399
ENSG00000274021	*RP11-359K18.4*		-1.8323906	0.04978705
ENSG00000170426	*FRG1JP*	short chain dehydrogenase/reductase family 9C, member 7	-1.8252158	0.04854333
ENSG00000214814	*FER1L6*	fer-1-like family member 6	-1.8183546	0.02259699
ENSG00000131620	*ANO1*	anoctamin 1, calcium activated chloride channel	-1.775734	0.03580051
ENSG00000162998	*FRZB*	frizzled-related protein	-1.7598748	0.04041998
ENSG00000162949	*ARL10*	calpain 13	-1.7397321	0.04762765
ENSG00000151715	*TMEM45B*	transmembrane protein 45B	-1.639473	0.03906328
ENSG00000013016	*EHD3*	EH-domain containing 3	-1.625314	0.04086461

ENSG00000196872	*CREB5*	KIAA1211-like	-1.6019891	0.04775302
ENSG00000231674	*SLC25A24*	long intergenic non-protein coding RNA 410	-1.5980658	0.04344484
ENSG00000267197	*LRRC39*		-1.5980658	0.04440034
ENSG00000185352	*RP11-20B7.1*	heparan sulfate 6-O-sulfotransferase 3	-1.5902457	0.04918476
ENSG00000175793	*AGPS*	stratifin	-1.5754469	0.0487429
ENSG00000142973	*ITGAD*	cytochrome P450, family 4, subfamily B, polypeptide 1	-1.5138524	0.0491994
ENSG00000187987	*RBL1*	zinc finger and SCAN domain containing 23	1.50382052	0.04902441
ENSG00000080007	*RP11-10O17.3*	DEAD (Asp-Glu-Ala-Asp) box polypeptide 43	1.50819184	0.04968186
ENSG00000204802	*GLRB*		1.51097515	0.04704692
ENSG00000227671	*SDR9C7*		1.52034242	0.04849889
ENSG00000018510	*DNAJB5-AS1*	alkylglycerone phosphate synthase	1.52087463	0.04879267
ENSG00000157827	*FMNL2*	formin-like 2 18267]	1.52906358	0.0344828
ENSG00000108932	*SLC16A6*	solute carrier family 16, member 6	1.55532917	0.03647016
ENSG00000100523	*DDHD1*	DDHD domain containing 1	1.55790743	0.03000496
ENSG00000215548	*LINC01224*	FSHD region gene 1 family member J, pseudogene	1.56392389	0.04866068
ENSG00000204837	*BHLHE41*		1.57435602	0.04641574
ENSG00000261115	*TMEM178B*	transmembrane protein 178B	1.6093989	0.02407209
ENSG00000189057	*FAM111B*	family with sequence similarity 111, member B	1.64040874	0.02418023
ENSG00000179242	*RP11-204M4.2*	cadherin 4, type 1, R-cadherin (retinal)	1.6566309	0.04641162
ENSG00000261775	*CH507-236L23.2*		1.65767634	0.04977134
ENSG00000280330	*CH507-338C24.2*		1.65767634	0.04977134
ENSG00000279177	*AC062028.1*		1.65767634	0.04977134
ENSG00000123095	*RP11-539L10.2*	basic helix-loop-helix family, member e41	1.69861877	0.04645286
ENSG00000153132	*CLGN*	calmegin	1.70061329	0.01349185
ENSG00000146409	*SLC18B1*	solute carrier family 18, subfamily B, member 1	1.71082183	0.03080595
ENSG00000162694	*CAMK1G*	exostosin-like glycosyltransferase 2	1.72256847	0.04767342
ENSG00000154310	*TNIK*	TRAF2 and NCK interacting kinase	1.72747256	0.01915633
ENSG00000170558	*CDH2*	cadherin 2, type 1, N-cadherin (neuronal)	1.74292957	0.0242855
ENSG00000139354	*GAS2L3*	growth arrest-specific 2 like 3	1.75646319	0.0263216

ENSG00000162878	*PKDCC*	protein kinase domain containing, cytoplasmic	1.76751276	0.02698816
ENSG00000164764	*SBSPON*	somatomedin B and thrombospondin, type 1 domain containing	1.7683902	0.03511089
ENSG00000057252	*SOAT1*	sterol O-acyltransferase 1	1.78004216	0.02476
ENSG00000106483	*CYTL1*	secreted frizzled-related protein 4	1.78606984	0.04954055
ENSG00000226751	*DNASE2B*		1.81861131	0.04912248
ENSG00000057019	*DCBLD2*	discoidin, CUB and LCCL domain containing 2	1.82368976	0.04270698
ENSG00000280145	*RP11-317P15.4*		1.83139213	0.04749352
ENSG00000168491	*CCDC110*	coiled-coil domain containing 110	1.85044688	0.01741206
ENSG00000131711	*MAP1B*	microtubule-associated protein 1B	1.85251643	0.01232065
ENSG00000189007	*ADAT2*	adenosine deaminase, tRNA-specific 2	1.86044352	0.0354144
ENSG00000108960	*MMD*	monocyte to macrophage differentiation-associated	1.87009619	0.01750723
ENSG00000114455	*HHLA2*	HERV-H LTR-associating 2	1.87183289	0.01211896
ENSG00000164649	*RP11-411K7.1*	cell division cycle associated 7-like	1.87294508	0.04475613
ENSG00000162520	*SYNC*	syncoilin, intermediate filament protein	1.89119771	0.0250393
ENSG00000253910	*PCDHGB2*	protocadherin gamma subfamily B, 2	1.93251717	0.00864516
ENSG00000176401	*SIGLEC8*	EP300 interacting inhibitor of differentiation 2B	1.93251717	0.04999554
ENSG00000166130	*IKBIP*	IKBKB interacting protein	1.93455736	0.03767763
ENSG00000183722	*LHFP*	lipoma HMGIC fusion partner	1.941342	0.02029464
ENSG00000188060	*RAB42*	RAB42, member RAS oncogene family	1.97288638	0.02559835
ENSG00000279312	*CH507-154B10.1*		1.98085801	0.04738529
ENSG00000135919	*SERPINE2*	serpin peptidase inhibitor, clade E (nexin, plasminogen activator inhibitor type 1), member 2	2.00924912	0.02550524
ENSG00000260837	*SHOX2*		2.04514306	0.04979501
ENSG00000168779	*AC011718.2*	short stature homeobox 2	2.04514306	0.04979501
ENSG00000272788	*PCDH1*		2.10127695	0.04885545
ENSG00000275318	*SH2D1B*		2.10127695	0.04926852
ENSG00000143469	*SYT14*	synaptotagmin XIV	2.11666541	0.02834196
ENSG00000198796	*ALPK2*	alpha-kinase 2	2.14609936	0.02160741
ENSG00000274776	*OR2A4*		2.16564711	0.04695039

ENSG00000156886	*RP11-526D8.11*	integrin, alpha D	2.28847471	0.04921033
ENSG00000240694	*PNMA2*	paraneoplastic Ma antigen 2	2.31943911	0.02067334
ENSG00000180658	*RP11-111F5.4*	olfactory receptor, family 2, subfamily A, member 4	2.32266201	0.04697349
ENSG00000197769	*MAP1LC3C*	microtubule-associated protein 1 light chain 3 gamma	2.33075048	0.00382904
ENSG00000109099	*PMP22*	peripheral myelin protein 22	2.33952201	0.00434329
ENSG00000162692	*VCAM1*	vascular cell adhesion molecule 1	2.35494248	0.02198844
ENSG00000146070	*PLA2G7*	phospholipase A2, group VII (platelet-activating factor acetylhydrolase, plasma)	2.40751244	0.03509484
ENSG00000120457	*KCNJ5*	potassium channel, inwardly rectifying subfamily J, member 5	2.41196055	0.02629045
ENSG00000170891	*LINC01010*	cytokine-like 1	2.43016017	0.04961189
ENSG00000274044	*RPS16*		2.51158596	0.0498035
ENSG00000137976	*CNTN5*	deoxyribonuclease II beta	2.56199743	0.04912422
ENSG00000143387	*CTSK*	cathepsin K	2.65689924	0.02031711
ENSG00000159674	*SPON2*	spondin 2, extracellular matrix protein	2.65890622	0.01281521
ENSG00000109738	*RP11-1036E20.9*	glycine receptor, beta	2.66322046	0.04719797
ENSG00000122254	*HS3ST2*	heparan sulfate (glucosamine) 3-O-sulfotransferase 2	2.68914533	0.00578757
ENSG00000113249	*HAVCR1*	hepatitis A virus cellular receptor 1	2.72888146	0.02351189
ENSG00000136235	*GPNMB*	glycoprotein (transmembrane) nmb	2.93831779	0.01096223
ENSG00000236700	*DDX43*	long intergenic non-protein coding RNA 1010	3.04690012	0.04963814
ENSG00000158270	*COLEC12*	collectin sub-family member 12	3.09694574	0.0128705
ENSG00000107593	*PKD2L1*	polycystic kidney disease 2-like 1	3.14517114	0.00371346
ENSG00000113361	*CDH6*	cadherin 6, type 2, K-cadherin (fetal kidney)	3.23217055	0.00565568
ENSG00000130208	*APOC1*	apolipoprotein C-I	3.51725819	0.03333679

2. Supplemental Figures

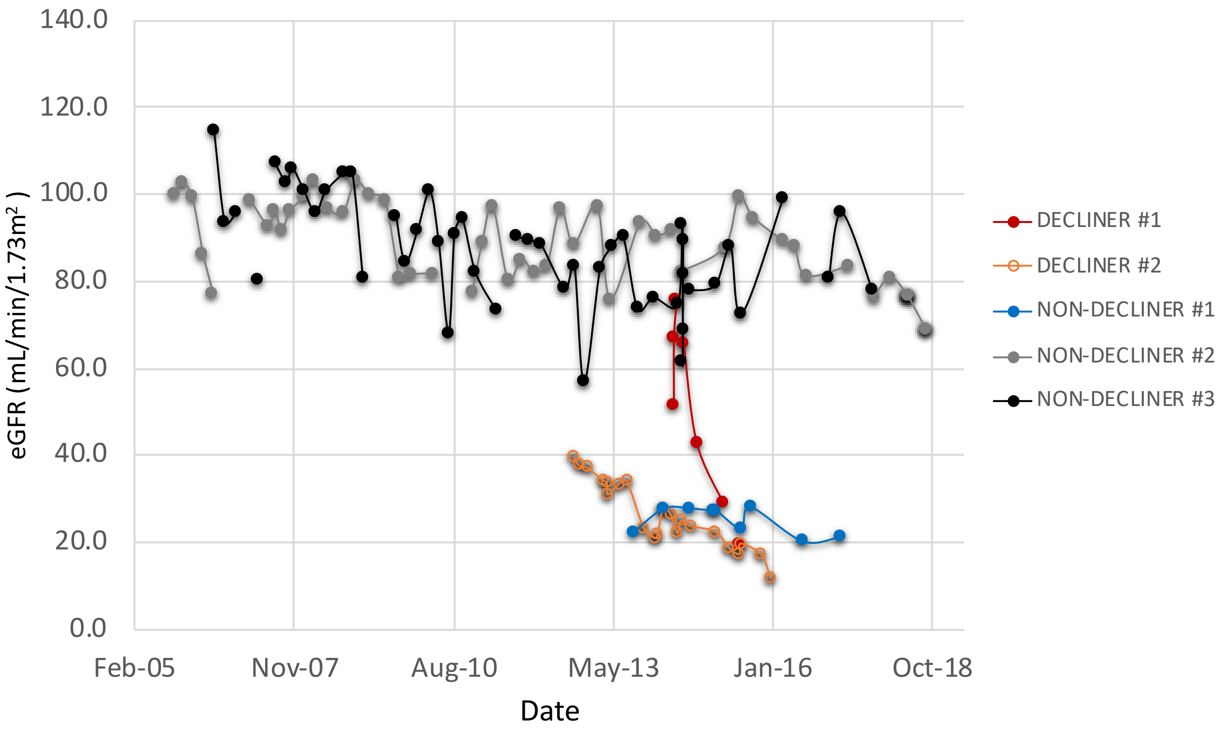

Supplemental Figure 1. Estimated glomerular filtration rate (eGFR) evolution of the type 1 diabetes patients selected for the transcriptomics study.

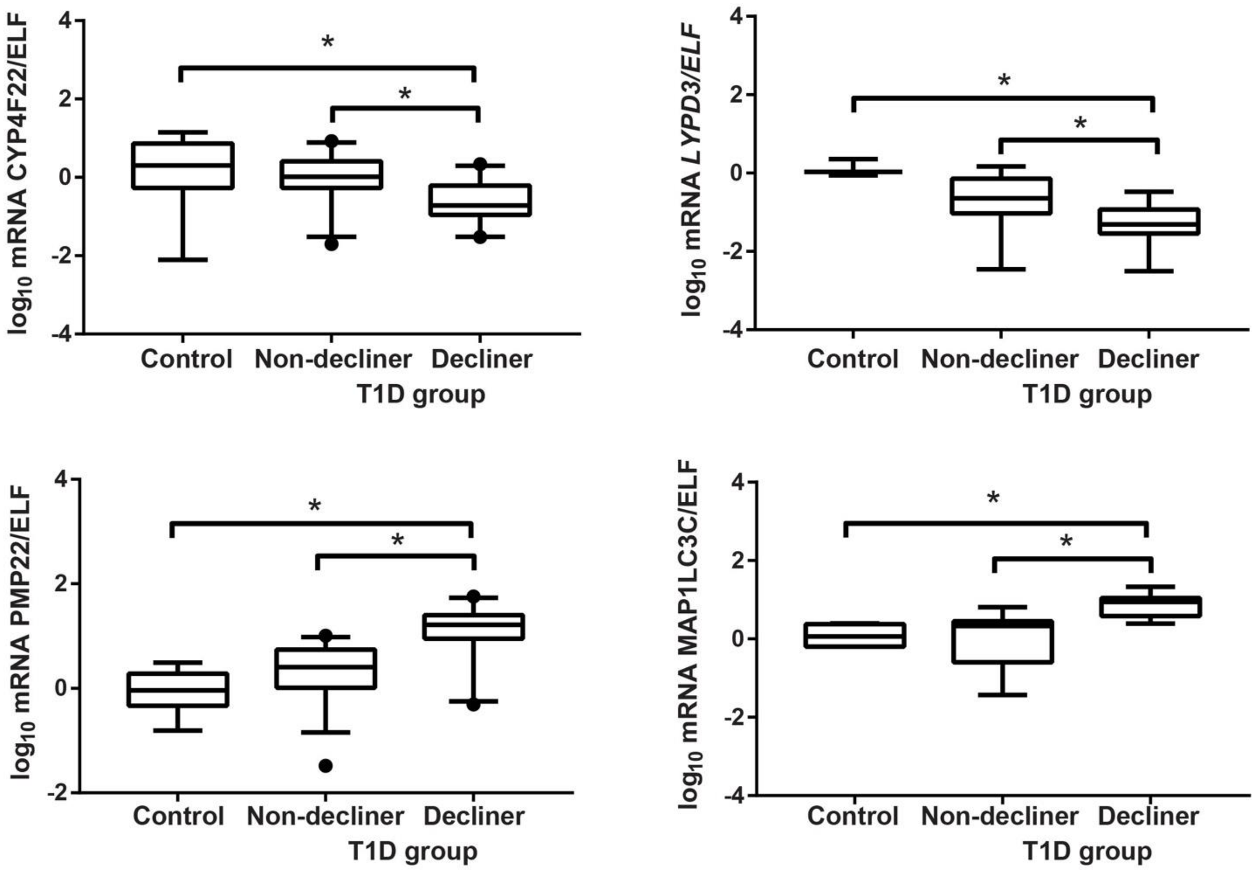

Supplemental Figure 2. Four genes differentially expressed in non-diabetic controls and type 1 diabetes (T1D) patients classified as non-decliners or decliners. Cross-sectional validation of genes differentially expressed in the human urinary sediment cells from non-diabetic controls and T1D patients classified as non-decliners or decliners (eGFR < or ≥3.5mL/min/1.73m2 per year of follow up, respectively). The horizontal lines within boxplots represent 5-95 percentile. **P*< 0.05

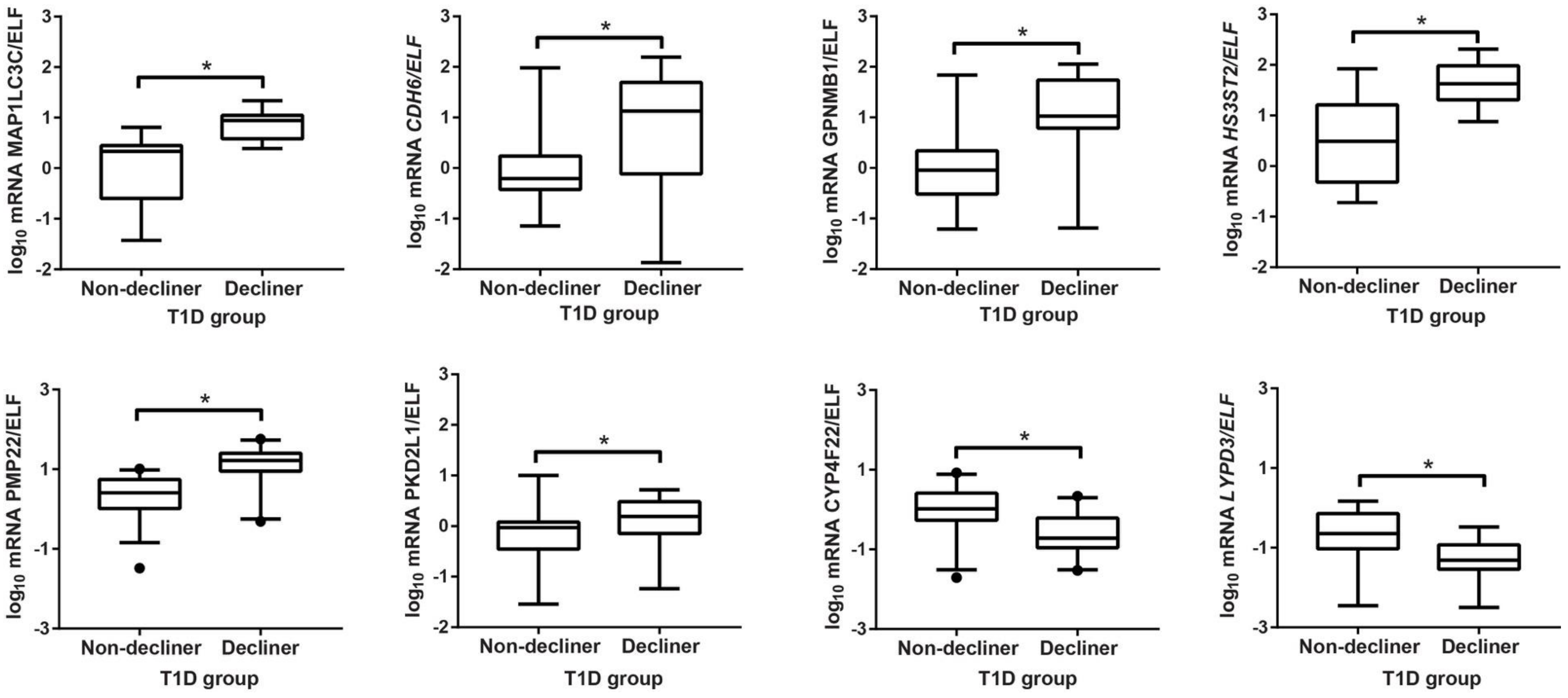

Supplemental Figure 3. Eight genes differentially expressed between type 1 diabetes (T1D) patients classified as non-decliners or decliners without adjustment for possible confounders. Cross-sectional validation of genes differentially expressed in the human urinary sediment cells from T1D patients classified as non-decliners or decliners (eGFR < or ≥3.5mL/min/1.73m2 per year of follow up, respectively). The horizontal lines within boxplots represent 5-95 percentile. *P< 0.05
